# *Candida albicans* in Oral Squamous Cell Carcinoma: From Microbial Dysbiosis to Tumor-Promoting Mechanisms and Translational Opportunities

**DOI:** 10.3390/ijms27146118

**Published:** 2026-07-08

**Authors:** Abdelhabib Semlali, Mohammed Al-Zharani, Manal Dahdah, Fatiha Chandad

**Affiliations:** 1 Groupe de Recherche en Écologie Buccale, Faculté de Médecine Dentaire, Université Laval, Quebec, QC G1V 0A6, Canada; manal.dahdah.1@ulaval.ca (M.D.); fatiha.chandad@greb.ulaval.ca (F.C.); 2 Biology Department, College of Science, Imam Mohammad Ibn Saud Islamic University (IMSIU), Riyadh 11623, Saudi Arabia; mmyalzahrani@imamu.edu.sa

**Keywords:** oral squamous cell carcinoma, *Candida albicans*, extracellular vesicules, virulence

## Abstract

Oral squamous cell carcinoma (OSCC) remains a major global health burden with limited improvement in survival rates. While traditional risk factors such as tobacco and alcohol are well established, increasing evidence highlights the role of the oral microbiome in carcinogenesis. Among microbial species, *Candida albicans* (*C. albicans*) has emerged as a potential contributor to tumor-promoting processes. Clinical studies consistently report increased fungal colonization in oral potentially malignant disorders and OSCC, with associations to disease severity and recurrence. Mechanistically, *C. albicans* contributes to carcinogenesis through acetaldehyde production, chronic inflammation, oxidative stress, epithelial signaling modulation, and extracellular vesicle (EV)-mediated communication. These pathways promote tumor microenvironment remodeling and epithelial transformation. However, conflicting evidence exists regarding causality, suggesting that fungal colonization may also result from tumor-associated ecological changes. From a translational perspective, *C. albicans* and EV-associated signatures may represent promising biomarkers and therapeutic targets, although further validation is required. This review highlights the emerging role of fungal–host interactions in OSCC and underscores their potential in microbiome-informed precision oncology.

## 1. Introduction

Oral squamous cell carcinoma (OSCC) accounts for more than 90% of oral malignancies and represents a major global health burden, with approximately 377,000 new cases and over 177,000 deaths reported annually worldwide [1]. Despite advances in surgery, radiotherapy, and systemic therapies, the 5-year survival rate of OSCC has improved only marginally over recent decades, largely due to late diagnosis, tumor recurrence, and therapeutic resistance [2]. While established risk factors such as tobacco use, alcohol consumption, and human papillomavirus (HPV) infection contribute significantly to OSCC pathogenesis, they do not fully explain disease incidence, heterogeneity, or progression, suggesting that additional biological contributors may be involved [3]. Increasing evidence indicates that the oral microbiome plays a critical role in regulating oral epithelial homeostasis and carcinogenesis [4]. The oral cavity harbors one of the most diverse microbial ecosystems in the human body, comprising more than 700 bacterial species in addition to fungi, viruses, archaea, and protozoa [5]. Dysbiosis of this complex ecosystem has been associated with oral potentially malignant disorders (OPMDs) and OSCC, supporting a functional link between microbial imbalance and tumorigenesis [6].

Microorganisms can contribute to carcinogenesis through multiple mechanisms, including induction of chronic inflammation, the production of carcinogenic metabolites, the modulation of host immune responses, and the direct alteration of epithelial signaling pathways. Among fungal species, *C. albicans* is the most prevalent fungal colonizer of the oral cavity. Although commonly present as a commensal organism in healthy individuals, *C. albicans* exhibits remarkable phenotypic plasticity and virulence potential, allowing it to transition from harmless colonization to pathogenic behavior under permissive conditions such as immune dysfunction, epithelial damage, or microbial dysbiosis [7,8]. This opportunistic pathogen possesses multiple virulence traits, including biofilm formation, secretion of hydrolytic enzymes, and production of carcinogenic metabolites, which enable its sustained interaction with host tissues.

Clinical and experimental studies increasingly support a link between *C. albicans* colonization and oral carcinogenesis. Elevated fungal carriage has been consistently observed in patients with OPMDs and OSCC, with fungal load positively correlating with disease severity and progression [7,9,10]. Mechanistically, *C. albicans* contributes to carcinogenesis through the production of carcinogenic metabolites such as acetaldehyde, the induction of chronic inflammation and oxidative stress, the modulation of epithelial signaling pathways, and EVs-mediated communication that reshapes the tumor microenvironment [11,12].

A narrative literature review approach was used to identify relevant studies related to *Candida albicans*, oral squamous cell carcinoma (OSCC), EVs, biofilms, and tumor microenvironment remodeling. Literature searches were conducted using PubMed, Scopus, and Web of Science databases, with emphasis placed on mechanistic, translational, and microbiome-focused studies.

This review provides a narrative synthesis of the literature focusing on mechanistic and translational aspects of *C. albicans* in oral carcinogenesis. We integrate clinical, molecular, and microbiological evidence to examine how fungal virulence, biofilm interactions, immune modulation, and EVs-mediated communication contribute to tumor initiation and progression. We further discuss emerging translational implications, including the potential of fungal biomarkers and targeted microbiome-based therapeutic strategies to improve early detection and clinical outcomes in OSCC. Despite increasing interest in the role of *Candida albicans* in OSCC, the available literature remains fragmented. Most previous reviews have primarily focused on individual mechanisms such as acetaldehyde production, inflammation, or microbial dysbiosis. However, an integrated framework linking clinical observations, fungal virulence, tumor microenvironment remodeling, extracellular vesicle-mediated communication, and translational implications is still lacking. This review aims to provide such an integrated perspective while critically evaluating current evidence supporting the concept of *Candida albicans* as a biological amplifier of oral carcinogenesis.

## 2. Biological Features of *C. albicans* Relevant to Oral Carcinogenesis

### 2.1. C. albicans as a Commensal and Opportunistic Pathogen

*C. albicans* is a commensal fungal species colonizing the oral cavity in approximately 30–60% of healthy individuals. Under physiological conditions, its growth is tightly regulated by host immune surveillance, epithelial barrier integrity [13,14], and microbial competition within the oral microbiome. However, disruption of this homeostatic balance through factors such as immunosuppression, epithelial damage, dysbiosis, or environmental stress can promote the transition of *C. albicans* from a commensal organism to an opportunistic pathogen [15]. A key feature of *C. albicans* biology is its morphological plasticity, characterized by reversible transitions between yeast, pseudohyphal, and hyphal forms [16,17]. This transition is critical for pathogenicity, as hyphal forms are associated with epithelial invasion, tissue damage, and activation of host inflammatory responses [18]. In addition, *C. albicans* forms structured biofilms on oral surfaces, providing protection from host defenses and antifungal agents while enabling persistent colonization. Importantly, *C. albicans* does not act in isolation but exists within polymicrobial communities. Interactions with oral bacteria contribute to biofilm stability, metabolic cooperation, and enhanced virulence, thereby sustaining prolonged host–microbe interactions [18,19]. These ecological and host-dependent characteristics position *C. albicans* as a microorganism capable of adapting to altered oral environments frequently observed in OPMDs and OSCC.

### 2.2. Morphological Plasticity and Adaptation to the Oral Environment

One of the most distinctive biological features of *C. albicans* is its remarkable morphological plasticity, which enables rapid adaptation to diverse environmental conditions within the oral cavity. Unlike many fungal species, *C. albicans* can reversibly transition between yeast, pseudohyphal, and hyphal forms in response to changes in temperature, pH, nutrient availability, oxygen tension, and host-derived signals [20,21]. This morphological flexibility plays a central role in fungal survival, colonization, and pathogenicity [22,23]. The yeast form is generally associated with commensal colonization and dissemination across mucosal surfaces, whereas the filamentous pseudohyphal and hyphal forms are more frequently linked to tissue invasion and pathogenic behavior [24]. Pseudohyphae represents an intermediate morphological state characterized by elongated cells that remain partially attached following cell division. In contrast, true hyphae are highly polarized filamentous structures capable of penetrating epithelial tissues and interacting directly with host cells [18,19].

The ability to switch between these morphological states provides *C. albicans* with a considerable adaptive advantage within the dynamic oral environment. Under physiological conditions, host immune surveillance, epithelial barrier integrity, and microbial competition help maintain fungal colonization in a commensal state. However, environmental disturbances such as dysbiosis, epithelial injury, inflammation, immunosuppression, or alterations in nutrient availability may trigger hyphal development and increased virulence [15,19].

Morphological transitions are closely associated with the expression of multiple virulence determinants, including adhesins, hydrolytic enzymes, biofilm-associated factors, and the pore-forming toxin candidalysin [21,23]. These factors collectively enhance fungal adhesion, epithelial invasion, tissue damage, and host inflammatory responses. Consequently, morphological plasticity not only promotes fungal persistence but also influences the intensity and duration of host–microbe interactions.

**Figure 1:**
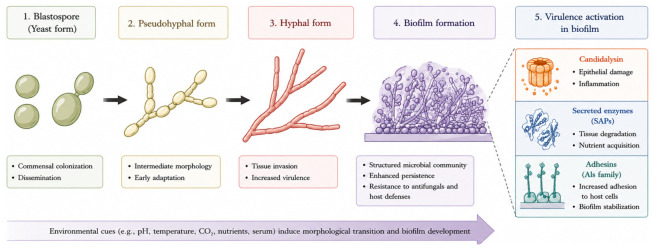
**Simplified representation of the major morphological transitions and virulence-associated traits of *C. albicans***. *C. albicans* is a versatile fungal pathogen capable of switching between yeast and hyphal forms, forming resilient biofilms, and expressing multiple virulence factors that facilitate colonization, persistence, and damage to the host. Created using BioRender.com (Abdelhabib Semlali, 2026. https://app.biorender.com/illustrations/canvas-beta/6a43ff8fdf9382c5d9624ef6, accessed on 20 June 2026) and finalized by the authors.

Hyphal formation is considered one of the major virulence-associated traits of *C. albicans*, facilitating epithelial invasion and persistent host interactions. Through direct tissue penetration and activation of inflammatory signaling pathways, hyphal structures contribute to prolonged epithelial stress and microenvironmental alterations that may become relevant in chronic oral diseases and potentially in oral carcinogenesis. The major morphological forms of *C. albicans* and their biological significance are summarized in Figure 1.

### 2.3. Biofilm Formation and Polymicrobial Interactions

A major factor contributing to the persistence and pathogenicity of *C. albicans* in the oral cavity is its ability to form highly organized biofilms on both biotic and abiotic surfaces [20]. Biofilms are structured microbial communities embedded within a self-produced extracellular matrix composed of polysaccharides, proteins, lipids, and extracellular nucleic acids [24,25]. This matrix provides mechanical stability, facilitates nutrient exchange, and protects microbial cells from environmental stressors, host immune defenses, and antimicrobial agents.

Biofilm development is a dynamic and multistep process involving initial adhesion, cellular proliferation, maturation, and eventual dispersion. During maturation, *C. albicans* undergoes extensive morphological differentiation, generating complex three-dimensional structures composed of yeast cells, pseudohyphae, and hyphae embedded within the extracellular matrix [26]. These mature biofilms exhibit enhanced resistance to antifungal therapies and contribute to long-term fungal persistence within the oral cavity. Importantly, *C. albicans* rarely exists as a single-species organism in the oral environment. Instead, it participates in complex polymicrobial communities involving numerous bacterial species commonly associated with oral health and disease. Interactions between *C. albicans* and oral bacteria can be cooperative or competitive, influencing microbial composition, biofilm architecture, metabolic activity, and virulence potential [18,19]. Several bacterial species have been shown to enhance fungal adhesion, hyphal development, and biofilm maturation, while fungal metabolic activity may reciprocally influence bacterial colonization and community stability [27]. These polymicrobial interactions are increasingly recognized as important contributors to oral dysbiosis. Rather than acting independently, fungal and bacterial communities establish ecological networks that can modify the local microenvironment through nutrient competition, metabolite production, inflammatory stimulation, and immune modulation [28,29]. Such interactions may promote persistent microbial colonization and prolonged host–microbe communication, creating conditions favorable for chronic mucosal inflammation and epithelial stress [30].

From a pathological perspective, biofilms serve as reservoirs of virulence-associated molecules, including hydrolytic enzymes, inflammatory mediators, and fungal metabolites capable of interacting with host tissues [31]. Persistent biofilm-associated colonization has been linked to sustained epithelial damage, increased inflammatory responses, and alterations in tissue homeostasis. Although the direct contribution of fungal biofilms to oral carcinogenesis remains incompletely understood, their ability to maintain chronic inflammatory and dysbiotic conditions provides a biologically plausible mechanism through which *C. albicans* may influence disease progression.

**Figure 2:**
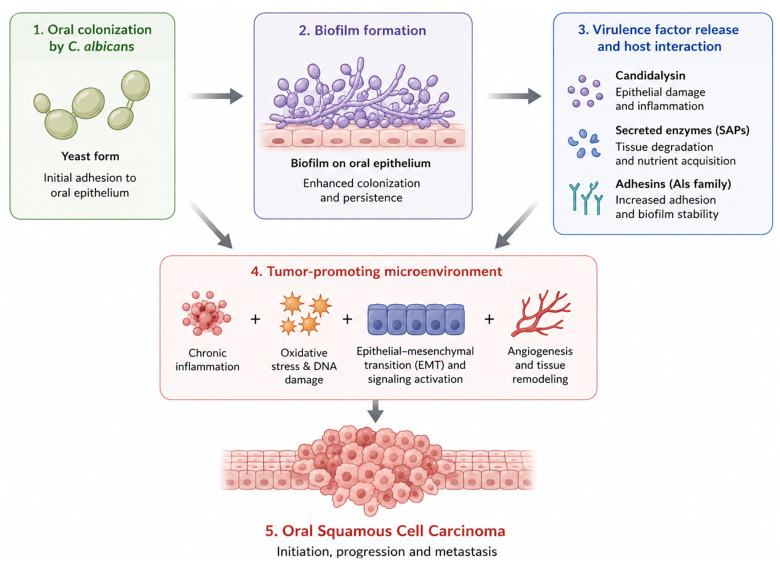
***C. albicans*-driven mechanisms contributing to oral squamous cell carcinoma**. Created using BioRender.com (Abdelhabib Semlali, 2026. https://app.biorender.com/illustrations/canvas-beta/6a43ff8fdf9382c5d9624ef6, accessed on 20 June 2026) and finalized by the authors.

Collectively, the capacity of *C. albicans* to form resilient biofilms and engage in complex polymicrobial interactions represents a critical aspect of its biology. These ecological and pathogenic properties facilitate long-term persistence within the oral cavity and establish a microenvironment characterized by chronic host–microbe interactions, which may contribute to the development and progression of oral diseases, including OSCC (Figure 2).

## 3. Clinical Evidence Linking *C. albicans* to Oral Carcinogenesis: Strengths, Limitations, and Causality Considerations

### 3.1. Increased Prevalence of C. albicans in OPMDs and OSCC

**Figure 3:**
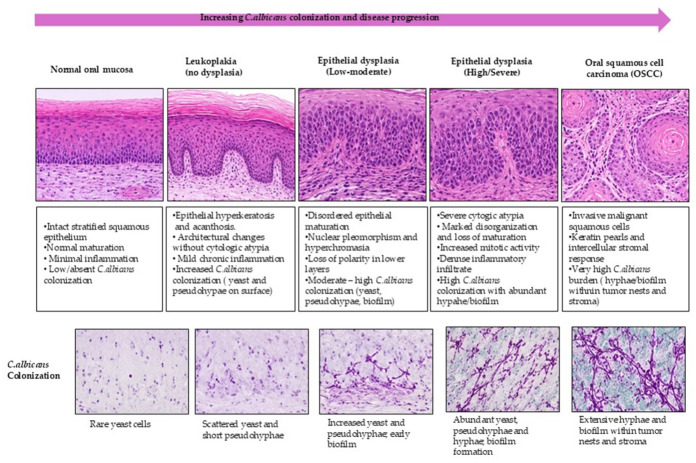
**Clinical association between *C. albicans* colonization and oral potentially malignant disorders (OPMDs)**. The schematic illustrates the progressive transition from normal oral mucosa to leukoplakia, epithelial dysplasia, and OSCC in association with increasing *C. albicans* colonization and dysbiosis. The arrows indicate the progressive increase in *C. albicans* colonization and disease severity from normal oral mucosa to OSCC. Figure created using BioRender.com (Abdelhabib Semlali, 2026. https://app.biorender.com/illustrations/canvas-beta/6a43ff8fdf9382c5d9624ef6, accessed on 20 June 2026) and finalized by the authors.

A substantial body of clinical evidence has demonstrated increased oral colonization by *C. albicans* in patients with oral potentially malignant disorders (OPMDs) and oral squamous cell carcinoma (OSCC) compared with healthy individuals [32,33,34]. Early studies reported associations between *C. albicans* colonization and oral leukoplakia, suggesting a possible role in malignant transformation [21]. Subsequent investigations confirmed that *C. albicans* is the predominant fungal species isolated from dysplastic lesions and oral cancers [7,24]. Prevalence rates in OSCC patients range from 60% to over 80%, depending on the population and detection methods used [9,25,26]. Importantly, quantitative studies indicate that fungal burden correlates with the severity of epithelial dysplasia, supporting a dose-dependent relationship between *C. albicans* colonization and disease progression [8,28]. In addition, increased expression of virulence-associated genes, including candidalysin-encoding genes, has been reported in isolates from OSCC lesions, suggesting enhanced pathogenic potential [29]. These findings support a strong association between *C. albicans* colonization and oral carcinogenesis. However, association alone does not establish causality, and interpretation requires careful consideration of study design and confounding factors. The principal clinical studies supporting these observations are summarized in Table 1, while the progressive increase in fungal colonization during disease evolution is illustrated in Figure 3.

### 3.2. Strain-Level Heterogeneity and High-Risk Phenotypes

Emerging evidence indicates that *C. albicans* exhibits significant strain-level heterogeneity, with important implications for carcinogenic potential. Not all strains display equivalent virulence, metabolic activity, or ability to modulate host responses. Clinical isolates obtained from OSCC patients have been shown to produce higher levels of acetaldehyde compared with commensal strains, suggesting enhanced genotoxic capacity [10,31,35]. Similarly, variability in biofilm formation, hyphal transition, and expression of virulence factors such as candidalysin may contribute to differences in epithelial damage and inflammatory responses [36,37,38]. These strain-dependent differences are biologically relevant because EV cargo directly influences host–pathogen communication. Virulence-associated proteins, secreted enzymes, regulatory RNAs, lipids, and metabolites transported by EVs can differentially modulate epithelial signaling pathways, inflammatory cytokine production, immune cell activation, oxidative stress responses, and extracellular matrix remodeling. Consequently, highly virulent *C. albicans* strains may produce EVs with greater tumor-promoting potential by enhancing chronic inflammation, facilitating epithelial damage, and promoting immune evasion within the OSCC microenvironment [28,39,40]. These findings suggest that specific high-risk *C. albicans* phenotypes may disproportionately contribute to tumor-promoting processes. However, current clinical studies rarely incorporate strain-level or functional characterization, representing a significant limitation in understanding the biological relevance of fungal heterogeneity in OSCC.

### 3.3. Association with Disease Progression, Severity, and Recurrence

Beyond increased prevalence, several studies report associations between *C. albicans* colonization and adverse clinical outcomes. Higher fungal burden has been observed in advanced tumor stages, suggesting a possible link between fungal persistence and tumor progression [9]. Persistent *Candida* colonization has also been associated with increased risk of lesion progression and recurrence [24,26]. Furthermore, patients with chronic *Candida*-associated conditions, such as autoimmune polyendocrinopathy-candidiasis-ectodermal dystrophy (APECED), exhibit increased susceptibility to squamous cell carcinomas, providing additional clinical support for a link between persistent fungal infection and carcinogenesis [28]. Nevertheless, these observations remain correlative. The directionality of this association remains unclear, as tumor-associated changes such as epithelial disruption, immune dysregulation, and altered salivary composition may themselves promote fungal colonization [3,41].

To facilitate comparison among published clinical studies, the principal investigations evaluating the association between *C. albicans* and oral potentially malignant disorders (OPMDs) or OSCC are summarized in Table 1. The table highlights differences in study design, patient populations, detection methods, principal findings, and study limitations, illustrating both the consistency of the reported associations and the methodological heterogeneity across studies.

**Table 1:** Summary of clinical studies investigating *C. albicans* in OPMDs and OSCC.

Study	Population	Patient Number	Lesion Type	Detection Method	Main Findings	Limitations
Krogh et al. [42]	Adults	*n* = 45	Leukoplakia	culture	Increased *Candida* prevalence in dysplastic lesions	Small cohort
Alnuaimi et al. [9]	OSCC patients	*n* = 72	OSCC	PCR/Culture	Higher fungal burden associated with a tumor severity	Cross-sectional
Bakri et al. [43]	OPMD patients	*n* = 60	Dysplasia	Oral Swab culture	*Candida* colonization correlated with dysplasia grade	No longitudinal follow-up

Overall, despite differences in study design and methodology, most investigations consistently reported increased *C. albicans.* colonization in OPMDs and OSCC. Nevertheless, the predominance of cross-sectional studies limits causal inference and highlights the need for well-designed longitudinal investigations.

### 3.4. Functional and Molecular Evidence Supporting Clinical Associations

**Figure 4:**
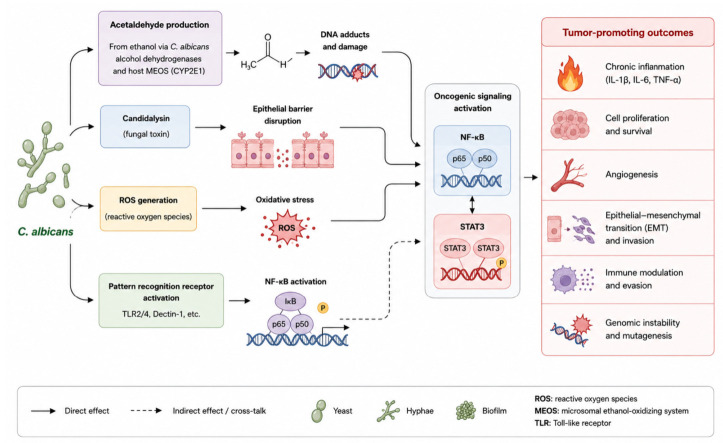
**Mechanistic cascade linking *C. albicans* to oral carcinogenesis**. Figure created using BioRender.com (Abdelhabib Semlali, 2026. https://app.biorender.com/illustrations/canvas-beta/6a43ff8fdf9382c5d9624ef6, accessed on 20 June 2026) and finalized by the authors.

Mechanistic and molecular studies provide biological plausibility supporting the clinical association between *C. albicans* and OSCC [34,44]. Elevated salivary acetaldehyde levels have been reported in patients with oral cancer, particularly in the context of alcohol exposure [10,45]. In addition, tumor-associated microbiome analyses reveal enrichment of *Candida* species within cancer tissues compared with adjacent non-tumor mucosa [41,46]. Experimental studies further demonstrate that fungal colonization leads to the release of virulence-associated factors, including acetaldehyde, secreted proteases, and EVs [47]. These fungal-derived molecules induce chronic epithelial stress and activate pro-inflammatory signaling pathways, resulting in sustained production of inflammatory cytokines such as IL-6, IL-8, and TNF-α through activation of pathways such as IL-17-mediated responses [48]. Chronic inflammation promotes the generation of reactive oxygen species (ROS), leading to oxidative DNA damage and genomic instability [49]. These virulence mechanisms activate inflammatory and oncogenic signaling pathways discussed later (Figure 4). Concurrently, *C. albicans* and associated polymicrobial biofilms contribute to tumor microenvironment remodeling by promoting immune dysregulation, inflammatory amplification, and cellular transformation [50]. These interconnected mechanisms collectively facilitate epithelial dysfunction, tumor progression, and the development of oral squamous cell carcinoma (OSCC). However, most of these studies are conducted in vitro or in preclinical models, and their direct translation to human disease remains limited.

### 3.5. Limitations of Clinical Studies and Causality Considerations

Despite consistent associations, several limitations prevent the definitive establishment of a causal relationship between *C. albicans* and oral carcinogenesis. Most clinical studies are observational in nature. However, most studies remain cross-sectional, limiting causal inference. As a result, it remains difficult to determine whether fungal colonization precedes tumor development or arises because of tumor-associated ecological changes. Confounding factors, including tobacco use, alcohol consumption, immunosuppression, and global microbiome dysbiosis, further complicate interpretation, as they may independently promote both fungal colonization and carcinogenesis [3,41]. In addition, tumor-associated microenvironmental changes, such as epithelial damage, reduced salivary flow, and immune dysfunction, may create permissive conditions for fungal overgrowth. These observations raise the possibility that *Candida* colonization may be, at least in part, a secondary phenomenon. Importantly, the absence of longitudinal studies represents a critical gap in the field. Establishing temporality is essential to determine whether *C. albicans* acts as an initiator, promoter, or bystander in oral carcinogenesis.

#### Mechanistic Contribution of Confounding Factors

Several established OSCC risk factors may simultaneously promote fungal persistence and carcinogenesis [50]. Tobacco and alcohol exposure can alter oral epithelial integrity, induce oxidative stress, and increase acetaldehyde production, thereby facilitating both *C. albicans* colonization and epithelial transformation [51]. Similarly, dysbiosis, xerostomia, immunosuppression, and epithelial injury create permissive ecological conditions that support chronic fungal persistence, inflammatory amplification, and tumor-promoting microenvironment remodeling [52].

### 3.6. Integrated Clinical Interpretation: From Association to Biological Amplification

Although numerous clinical and experimental studies support an association between *C. albicans* colonization and OSCC progression, current evidence remains insufficient to establish a direct causal relationship [13,14,25]. An important consideration is the possibility of reverse causation, whereby tumor-associated ecological and immunological changes may themselves promote fungal colonization rather than fungal colonization acting as the primary initiating event. OSCC-associated tissue damage, epithelial barrier disruption, hypoxia, altered salivary composition, immune dysregulation, and microbial dysbiosis may collectively create permissive ecological niches that facilitate persistent *C. albicans* colonization [53]. In this context, *C. albicans* may function not as a primary oncogenic driver but rather as a biological amplifier of tumor-promoting processes [54]. Persistent fungal colonization may exacerbate chronic inflammation, oxidative stress, epithelial injury, immune dysregulation, and tumor microenvironment remodeling, thereby accelerating disease progression once premalignant or malignant changes have already been initiated [55]. This conceptual framework may help reconcile the strong biological plausibility of fungal involvement with the current lack of definitive causal evidence. Importantly, this model is consistent with broader concepts emerging in microbiome-associated carcinogenesis, in which microbial communities contribute to tumor progression through ecological and inflammatory amplification rather than acting as isolated carcinogenic agents.

## 4. Mechanistic Insights into *C. albicans* Driven Oral Carcinogenesis

### 4.1. Carcinogenic Metabolites and Genotoxic Stress

One of the most well-established mechanisms linking *C. albicans* to oral carcinogenesis is its ability to metabolize ethanol into acetaldehyde, a recognized Group I carcinogen [56,57]. Acetaldehyde induces DNA damage through the formation of DNA adducts, chromosomal instability, and interference with DNA repair pathways [56,57]. In addition, *C. albicans* has been implicated in the formation of carcinogenic N-nitrosamines, further contributing to mutagenesis and genomic instability [58]. Importantly, clinical isolates from OSCC patients have been shown to produce higher levels of acetaldehyde compared to commensal strains, suggesting a strain-dependent carcinogenic potential [10]. Acetaldehyde accumulation within the oral cavity may result not only from fungal alcohol dehydrogenase activity but also from host-associated metabolic pathways, including the microsomal ethanol-oxidizing system (MEOS) mediated by cytochrome P450 2E1 (CYP2E1) [59]. In the presence of ethanol exposure, these complementary metabolic pathways may synergistically enhance local acetaldehyde production, thereby increasing epithelial genotoxic stress and carcinogenic potential [59,60]. Importantly, fungal acetaldehyde production appears to be highly dependent on ethanol availability, suggesting that chronic alcohol consumption may amplify the carcinogenic effects of *C. albicans* colonization [43]. In addition to acetaldehyde-mediated genotoxicity, candidalysin, a pore-forming peptide toxin secreted during hyphal formation, contributes directly to epithelial injury through membrane disruption, calcium influx, reactive oxygen species (ROS) generation, and activation of inflammatory signaling pathways [61]. Candidalysin-induced epithelial barrier disruption facilitates persistent microbial invasion and sustained inflammatory activation, thereby promoting a tumor-supportive microenvironment [62].

### 4.2. Chronic Inflammation and Oncogenic Signaling Pathways

Persistent colonization by *C. albicans* promotes chronic inflammation, a well-recognized enabling characteristic of cancer [36,63]. Fungal interaction with epithelial and immune cells leads to sustained production of pro-inflammatory cytokines, including IL-1β, IL-6, IL-8, and TNF-α [36,64]. These inflammatory mediators activate key oncogenic signaling pathways, particularly NF-κB and STAT3, which regulate epithelial proliferation, survival, angiogenesis, and resistance to apoptosis [63]. Chronic activation of these pathways creates a pro-tumorigenic environment that favors malignant transformation.

The OSCC microenvironment is characterized by profound immune remodeling that promotes tumor progression and immune evasion [65]. Accumulating evidence indicates that OSCC tissues exhibit increased infiltration of immunosuppressive cell populations, including regulatory T cells, myeloid-derived suppressor cells, and alternatively activated M2 macrophages [66]. These cells contribute to the suppression of effective anti-tumor immunity through the secretion of immunoregulatory cytokines and inhibition of cytotoxic T-cell activity. In parallel, chronic inflammatory signaling promotes T-cell dysfunction and exhaustion, extracellular matrix remodeling, angiogenesis, and stromal activation, collectively establishing a permissive microenvironment for tumor growth and metastasis [67]. Recent studies further emphasize the dynamic interactions between tumor cells, stromal components, and immune populations as central drivers of OSCC progression and therapeutic resistance [65]. Persistent *C. albicans* colonization may contribute to remodeling of the OSCC immune microenvironment through chronic inflammatory stimulation and immune dysregulation [50]. Sustained fungal exposure promotes recruitment and activation of tumor-associated immune cells, including neutrophils, macrophages, and dysfunctional T-cell populations [68]. Emerging evidence suggests that chronic fungal-driven inflammation may favor macrophage polarization toward tumor-supportive M2 phenotypes characterized by immunosuppressive and pro-angiogenic activity [69]. In parallel, prolonged inflammatory signaling may impair cytotoxic T-cell responses and promote T-cell exhaustion, thereby reducing effective anti-tumor immunity. These immune alterations contribute to the establishment of a permissive tumor microenvironment characterized by immune evasion, inflammatory amplification, and enhanced tumor progression [70]. Recent studies further indicate that stromal and immune components of the OSCC microenvironment actively interact with microbial-derived inflammatory signals, contributing to extracellular matrix remodeling, angiogenesis, and metastatic potential [66,71].

### 4.3. Oxidative Stress and Genomic Instability

In addition to inflammatory signaling, *C. albicans* contributes to carcinogenesis through the induction of oxidative stress. Infection leads to increased production of reactive oxygen species (ROS), derived from both fungal metabolism and host immune responses [63,72]. Elevated ROS levels induce oxidative DNA damage, lipid peroxidation, and genomic instability, all of which are central drivers of tumor initiation and progression [72,73]. Furthermore, candidalysin, a peptide toxin secreted by hyphal forms, exacerbates epithelial damage and promotes inflammatory and oxidative stress signaling [38,74].

### 4.4. Direct Modulation of Epithelial Signaling and Cellular Plasticity

Beyond indirect effects, *C. albicans* can directly modulate epithelial cell signaling pathways. Hyphal invasion activates mitogen-activated protein kinase (MAPK) pathways, including ERK, JNK, and p38, which regulate cellular proliferation and stress responses [75]. Experimental studies have demonstrated that *C. albicans* exposure induces epithelial–mesenchymal transition (EMT)-like changes, characterized by loss of epithelial markers, increased cellular motility, and enhanced invasive potential [63]. These changes are critical in tumor progression and metastasis. In addition, fungal interactions may promote resistance to apoptosis, allowing survival and expansion of genetically altered epithelial cells [74].

### 4.5. EVs-Mediated Cross-Kingdom Communication

Emerging evidence identifies EVs as key mediators of communication between *C. albicans* and host cells. Fungal EVs transport a diverse cargo, including virulence proteins, lipids, and regulatory RNAs [21,24,75,76,77]. Upon internalization by epithelial and immune cells, EVs activate inflammatory signaling pathways such as NF-κB and MAPK, leading to cytokine production and epithelial stress responses [11,12,35]. EV-associated molecules may also alter host gene expression, influencing proliferation, apoptosis resistance, and immune modulation. However, the direct causal contribution of fungal EVs to OSCC initiation remains to be fully established [78,79].

### 4.6. Integrated Mechanistic Model and Biological Amplification

**Figure 5:**
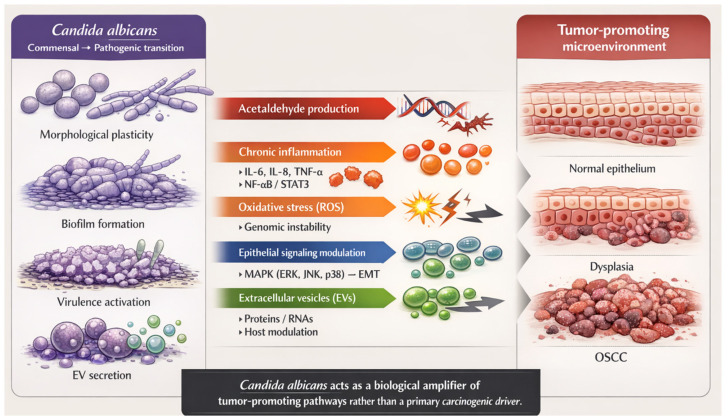
***C. albicans* contributes to oral carcinogenesis through cross-kingdom interactions involving acetaldehyde production, chronic inflammation, oxidative stress, epithelial signaling modulation, and extracellular vesicle-mediated communication**. Figure created using BioRender.com (Abdelhabib Semlali, 2026. https://app.biorender.com/illustrations/canvas-beta/6a43ff8fdf9382c5d9624ef6, accessed on 20 June 2026) and finalized by the authors.

Collectively, these mechanisms support a multistep model in which *C. albicans* acts as a biological amplifier of tumor-promoting processes. Through coordinated effects involving carcinogenic metabolite production, chronic inflammation, oxidative stress, epithelial signaling modulation, and EV-mediated communication, fungal colonization contributes to tumor microenvironment remodeling and epithelial transformation [11,12,35]. Nevertheless, conflicting evidence exists regarding the causal role of *C. albicans* in OSCC, as fungal colonization may also arise because of tumor-associated microenvironmental changes rather than acting as a primary driver [3,41]. An integrated overview of these cross-kingdom mechanisms is illustrated in Figure 5.

Collectively, these findings indicate that *C. albicans* may contribute to OSCC progression through interconnected mechanisms involving chronic inflammation, oxidative stress, epithelial signaling dysregulation, and extracellular vesicle-mediated communication. The overall strength of evidence supporting each proposed mechanism is summarized in Table 2.

The current level of evidence supporting the proposed tumor-promoting mechanisms is summarized in Table 2. This overview illustrates that while some mechanisms, such as acetaldehyde production and chronic inflammation, are supported by substantial experimental and clinical evidence, others, including extracellular vesicle-mediated communication, remain emerging areas of investigation.

This comparison emphasizes that the biological evidence supporting the involvement of *C. albicans* is heterogeneous. Consequently, future mechanistic studies are required to strengthen causal inference and better define the contribution of individual virulence mechanisms during OSCC progression.

Experimental studies investigating the molecular mechanisms linking *C. albicans* to oral carcinogenesis are summarized in Table 3. These studies collectively provide biological plausibility for the clinical associations observed in patients by demonstrating multiple tumor-promoting effects of fungal colonization under controlled experimental conditions.

Although these experimental findings provide valuable mechanistic insights, most studies were conducted using in vitro models or animal experiments. Therefore, their translation to human disease should be interpreted cautiously until validated in prospective clinical studies.

## 5. EVs as Emerging Mediators Linking *C. albicans* to Oral Carcinogenesis

EVs are membrane-enclosed nanoparticles released by virtually all cell types, including fungal, bacterial, and mammalian cells [87]. These vesicles transport diverse bioactive cargo such as proteins, lipids, metabolites, and regulatory RNAs capable of modulating recipient cell behavior [88]. Increasing evidence suggests that fungal EVs contribute to host–microbe communication and may represent an important mechanistic link between *C. albicans* colonization and oral carcinogenesis [88]. Representative experimental studies supporting the proposed biological amplification model are summarized in Table 3.

### 5.1. Biology and Biogenesis of Fungal EVs

*C. albicans* actively secretes EVs containing virulence-associated molecules, including adhesins, hydrolytic enzymes, lipids, heat shock proteins, and regulatory RNAs [11,77,89]. These vesicles can interact with oral epithelial and immune cells without requiring direct fungal invasion, thereby facilitating long-distance communication within the oral microenvironment. Experimental studies have demonstrated that fungal EVs contribute to epithelial stress responses, immune modulation, and barrier dysfunction [78,90]. EV-mediated signaling may amplify inflammatory and stress-related pathways previously discussed in this review, thereby promoting conditions favorable for chronic inflammation and tumor-supportive microenvironment remodeling [91].

A major challenge in fungal EV research is the difficulty in distinguishing fungal-derived vesicles from host-derived EVs in complex biological environments such as saliva and biofilm-associated samples [92]. In addition, EV cargo contamination resulting from co-isolated proteins, lipoproteins, and non-vesicular extracellular components may complicate the interpretation of functional studies [88]. The absence of robust fungal-specific EV markers further limits standardization and reproducibility across studies.

### 5.2. Role of Fungal EVs in Host–Microbe Communication

Within polymicrobial biofilms, EVs contribute to microbial communication, adaptation, and persistence. Fungal EVs may interact with bacterial outer membrane vesicles, facilitating coordinated signaling and enhancing pathogenic potential. These interactions promote epithelial stress, biofilm stability, and tumor-supportive microenvironmental changes [12,79].

Biofilm-associated EV signaling may further contribute to immune dysregulation, stromal remodeling, and persistent inflammatory activation, thereby supporting tumor progression and resistance to host defenses.

### 5.3. EV-Mediated Modulation of Epithelial and Immune Cell Function

**Figure 6:**
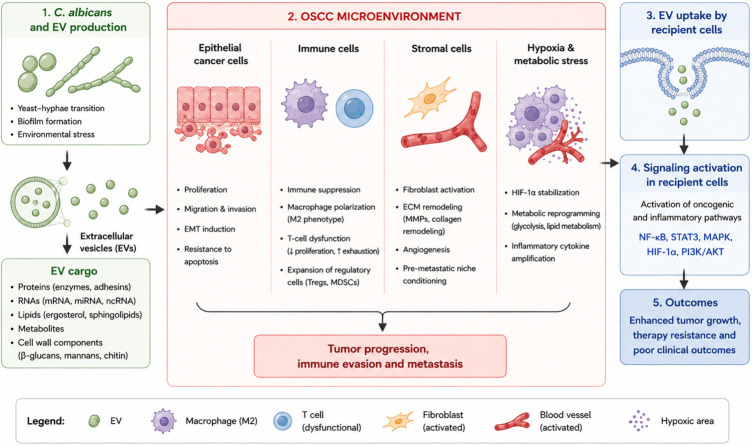
**EV-mediated cross-kingdom communication in oral carcinogenesis**. Figure created using BioRender.com (Abdelhabib Semlali, 2026. https://app.biorender.com/illustrations/canvas-beta/6a43ff8fdf9382c5d9624ef6, accessed on 20 June 2026) and adapted by the authors). *C. albicans* releases EVs containing virulence factors, proteins, lipids, and regulatory RNAs. These EVs interact with oral epithelial cells and immune cells, triggering inflammatory signaling pathways, including NF-κB activation and cytokine production. EV-mediated signaling promotes oxidative stress, epithelial dysfunction, and immune modulation. These processes contribute to tumor microenvironment remodeling and facilitate the development and progression of OSCC.

The involvement of EVs in fungal–host communication has important translational implications. Salivary EV-associated molecules represent promising candidates for non-invasive biomarkers reflecting fungal colonization, virulence activity, and tumor-associated microenvironmental changes [12,89]. However, current translational applications remain limited by methodological variability in EV isolation, lack of standardized fungal EV characterization protocols, and challenges related to cargo purity and fungal-versus-host EV discrimination [90]. In addition, targeting EV biogenesis, release, or uptake may represent a novel therapeutic strategy aimed at disrupting pathogenic communication networks without directly targeting microbial viability [91]. However, additional mechanistic and longitudinal studies are required before clinical translation can be fully achieved. Collectively, current evidence supports the concept that fungal EVs function as important mediators of cross-kingdom communication capable of influencing epithelial, immune, and microbial interactions within the OSCC microenvironment (Figure 6).

## 6. Translational Implications: Biomarkers, Prevention, and Therapeutic Opportunities

Current evidence suggests that *C. albicans* may contribute to oral carcinogenesis through multiple biological mechanisms, including chronic inflammation, acetaldehyde production, epithelial injury, oxidative stress, biofilm formation, and modulation of host immune responses [31,45,59,90]. However, most available data originate from in vitro studies, animal models, or cross-sectional clinical investigations, limiting definitive conclusions regarding causality [13,14,25].

From a clinical perspective, increased oral colonization by *C. albicans* has been consistently reported in oral potentially malignant disorders (OPMDs) and oral squamous cell carcinoma (OSCC) [15,18,23]. These observations raise the possibility that fungal burden, virulence-associated traits, or fungal-derived biomarkers could contribute to risk stratification and disease monitoring. Nevertheless, current evidence remains insufficient to support the routine clinical use of *Candida*-based biomarkers, and further validation in large prospective cohorts is required [13,14,25].

Therapeutically, modulation of oral fungal dysbiosis represents an attractive concept. Experimental studies suggest that reducing fungal burden or interfering with virulence-associated mechanisms such as hyphal formation, biofilm development, candidalysin production, and inflammatory signaling may attenuate tumor-promoting microenvironmental changes [31,38,45,90]. However, no clinical trials have yet demonstrated that antifungal interventions reduce the incidence or progression of OSCC [13,14].

Recent advances in extracellular vesicle (EV) research have also highlighted their potential role in host–fungal communication and disease progression [60,62,63]. Although fungal and host-derived EVs may eventually provide novel diagnostic or therapeutic opportunities, significant methodological challenges remain, including EV isolation variability, cargo contamination, limited fungal-specific markers, and lack of standardized analytical approaches [50,64]. Consequently, their clinical application remains exploratory.

Importantly, current evidence suggests a complex and bidirectional relationship between *C. albicans* colonization and OSCC progression. Tumor-associated ecological changes, including dysbiosis, immune dysfunction, epithelial damage, smoking, alcohol exposure, and xerostomia, may simultaneously facilitate fungal persistence and cancer progression [61,68,70,71]. This bidirectional relationship should be considered when interpreting clinical associations between *Candida* colonization and OSCC.

Future research should prioritize well-designed longitudinal studies, standardized in vitro and in vivo models, multi-omics investigations, and mechanistic analyses capable of distinguishing causal effects from secondary colonization phenomena [50,60,64]. Such approaches will be essential to determine whether *C. albicans* represents a modifiable risk factor, a biomarker of disease progression, or primarily a consequence of tumor-associated ecological alterations.

At present, the available evidence supports a role for *C. albicans* as a biological amplifier of oral carcinogenesis rather than a confirmed primary carcinogenic driver, highlighting the need for rigorous mechanistic and longitudinal studies to establish causality.

## 7. Conclusions and Future Perspectives

The relationship between *C. albicans* and oral squamous cell carcinoma (OSCC) reflects a complex and multifactorial interplay involving microbial ecology, host immune responses, and epithelial signaling pathways. Accumulating clinical, molecular, and experimental evidence supports the concept that *C. albicans* functions not merely as a passive commensal organism but as an active microbial modulator of tumor-promoting processes within the oral microenvironment. Multiple mechanistic pathways contribute to the potential role of *C. albicans* in oral carcinogenesis. These include the production of carcinogenic metabolites such as acetaldehyde, the induction of chronic inflammation, the generation of oxidative stress, the modulation of epithelial signaling pathways, and the disruption of immune homeostasis. Through these mechanisms, persistent fungal colonization may promote genomic instability, epithelial plasticity, and immune dysregulation, thereby lowering the threshold for malignant transformation. The ability of *C. albicans* to modulate immune and stromal components of the OSCC microenvironment further supports its role as a biological amplifier of tumor-promoting processes. Current evidence supports a model in which *C. albicans* acts primarily as a biological amplifier of carcinogenic processes rather than a direct oncogenic driver.

The integration of *C. albicans* into polymicrobial biofilms further amplifies its pathogenic potential. Within these structured microbial communities, fungal–bacterial interactions sustain chronic inflammatory signaling, enhance resistance to immune clearance, and contribute to tumor microenvironment remodeling. These ecological interactions highlight the importance of considering microbial communities, rather than individual pathogens, in understanding oral carcinogenesis. Importantly, emerging evidence identifies EVs as critical mediators of fungal–host communication. EVs derived from *C. albicans* and host cells can transport virulence factors, regulatory RNAs, and immunomodulatory molecules capable of influencing epithelial and immune cell behavior. These vesicle-mediated interactions represent a novel mechanistic dimension linking fungal persistence to tumor-promoting signaling and microenvironmental changes.

Although definitive causal relationships remain to be fully established, the convergence of epidemiological, molecular, and mechanistic data strongly supports a contributory role for *C. albicans* as a biological amplifier of carcinogenic processes rather than a primary oncogenic driver. This paradigm shift emphasizes the importance of microbial-host interactions in cancer biology and underscores the potential clinical relevance of fungal colonization in oral cancer risk and progression. Future research priorities should focus on longitudinal clinical studies to clarify causal relationships, the integration of multi-omics approaches to characterize fungal–host interactions, and the investigation of EV–mediated communication in tumor progression. In addition, the development of non-invasive diagnostic tools based on salivary fungal biomarkers and EV profiling may enable early detection and risk stratification in oral cancer. From a therapeutic perspective, targeting fungal biofilms, modulating oral microbiome composition, and disrupting EV-mediated signaling pathways represent promising strategies for reducing tumor-promoting microbial influences. Such approaches may complement existing cancer therapies and contribute to personalized and microbiome-informed treatment strategies.

**Conclusion**. The relationship between *C. albicans* and oral squamous cell carcinoma (OSCC) reflects a complex interplay among microbial dysbiosis, host immune responses, and epithelial signaling pathways. Accumulating evidence indicates that *C. albicans* may contribute to tumor-promoting processes through mechanisms involving chronic inflammation, oxidative stress, acetaldehyde production, epithelial damage, and tumor microenvironment remodeling. However, current evidence remains insufficient to establish a direct causal role in oral carcinogenesis, as fungal colonization may also arise because of tumor-associated ecological and immunological alterations. Available data support a model in which *C. albicans* functions as a biological amplifier of tumor-promoting processes rather than a confirmed primary carcinogenic driver. Future studies should prioritize longitudinal clinical investigations, standardized in vitro and in vivo models, rigorous extracellular vesicle characterization, and integrated multi-omics approaches to clarify causal relationships and identify clinically relevant biomarkers and therapeutic targets. Such efforts will be essential to determine whether fungal dysbiosis represents a modifiable risk factor, a biomarker of disease progression, or predominantly a consequence of OSCC-associated microenvironmental changes. Collectively, current evidence supports a paradigm shift in our understanding of the role of *C. albicans* in oral carcinogenesis. Rather than acting primarily as a direct carcinogenic agent, *C. albicans* appears to function as a dynamic biological amplifier that exploits the altered tumor microenvironment while simultaneously reinforcing tumor-promoting mechanisms. Future interdisciplinary studies, integrating microbiology, oncology, immunology, and multi-omics technologies, will be essential to validate this emerging concept and translate it into clinically relevant diagnostic and therapeutic strategies.

From a clinical perspective, patients with OPMDs and OSCC may benefit from routine assessment of oral fungal colonization, particularly when presenting with persistent oral candidiasis or extensive dysbiosis. Although current evidence does not support systematic antifungal therapy solely to prevent malignant progression, early diagnosis and appropriate management of oral candidiasis remain important components of comprehensive oral care. Future prospective clinical trials should evaluate whether reducing fungal burden or targeting specific virulence factors can improve treatment response, reduce recurrence, or enhance the quality of life of patients with OSCC. Ultimately, integrating fungal diagnostics, oral microbiome analysis, and anti-virulence approaches into multidisciplinary OSCC management may represent a promising avenue toward microbiome-informed precision oncology.

## Figures and Tables

**Table 2:** Evidence strength was classified according to the current level of experimental, translational, and clinical support available in the literature.

Mechanism	Evidence Type	Evidence-Strength Classification	Key Limitations	Key References
Acetaldehyde production	Clinical + in vitro	Strong	Confounding factors (alcohol, microbiome)	[7,27,44,45,46]
Chronic inflammation	Clinical + experimental	Strong	Non-specific mechanism	[11,48,49]
Oxidative stress (ROS)	Experimental	Moderate	Limited human validation	[50,51]
Epithelial signaling modulation	In vitro	Moderate	Lack of in vivo evidence	[14,15,49]
Extracellular vesicles (EVs)	Experimental	Emerging	Lack of standardization and clinical data	[22,29,30,53,54]
Biofilm interactions	Clinical + in vitro	Moderate	Complex polymicrobial interactions	[9,38,43,61,80]

**Table 3:** Summary of in vitro and experimental studies investigating mechanistic links between *C. albicans* and oral carcinogenesis.

Study	Experimental Model/Cell Line	*Candida* Exposure	Main Findings	Proposed Mechanism
Krogh et al. [42]	Human oral epithelial cells	*C. albicans* isolates from leukoplakia	Increased epithelial dysplasia-associated changes	Acetaldehyde production and epithelial injury
Mohd Bakri et al. [80]	Oral keratinocytes	Clinical *C. albicans* isolates	Increased fungal adherence and epithelial damage	Hyphal invasion and inflammation
Alnuaimi et al. [9]	Oral epithelial cells	*C. albicans* biofilms	Enhanced inflammatory cytokine production	IL-6, IL-8, TNF-α induction
Perera et al. [4]	Human oral epithelial cells	*C. albicans* infection	Increased acetaldehyde production and DNA damage	Genotoxic stress
Bertolini et al. [52]	Mouse oral mucosa and epithelial models	Chronic *C. albicans* colonization	Accelerated dysplasia and tumor progression	IL-17-dependent inflammation
Dwivedi et al [81].	Human oral keratinocytes	Hyphal *C. albicans*	Epithelial barrier disruption and ROS generation	Candidalysin-mediated toxicity
Verma et al. [82,83]	Oral epithelial cell cultures	*C. albicans* biofilm-conditioned medium	Increased oxidative stress and inflammatory activation	ROS and NF-κB activation
Vargas et al. [84]	Epithelial and immune cell co-culture	*C. albicans* EVs	Modulation of cytokine responses	EV-mediated host signaling
Recent EV studies [47,85,86]	Oral epithelial cells	Fungal EVs	Activation of inflammatory pathways and epithelial stress responses	NF-κB, MAPK and immune modulation

## Data Availability

The original contributions presented in this study are included in the article. Further inquiries can be directed to the corresponding author.
